# 20 years of Evidence‐Based Dentistry — How have our patients benefited?

**DOI:** 10.1002/cre2.155

**Published:** 2018-12-26

**Authors:** Asbjorn Jokstad

The string “medical evidence” [tw] on PubMed suggests that this term has been around for two centuries, while other permutations like “scientific evidence” appears in 1946, “evidence based” (without the hyphen) in 1949, “research evidence” in 1967, and “evidence‐based recommendations” (incidentally by an artificial intelligence software) emerge in a title from 1991. Also in 1991, the term *evidence‐based medicine* appears in the title of an editorial authored by Dr. Gordon Guyatt at McMaster University in Hamilton, Canada (Guyatt, 1991). While this short editorial emphasized a need to include evidence into clinical practice, a full paper published in 1992 rationalized why there is a need to change the medical education radically to prepare better future physicians to practice according to best evidence (Guyatt et al., 1992). The timing was perfect, because south of their border, their cousins had established the Agency for Healthcare Research and Quality to develop “evidence‐based guidelines” [tw] (Woolf, 1992), while in U.K., the opening of the UK Cochrane Centre in November 1992 marked the beginning of the Cochrane collaboration (Chalmers, Dickersin, & Chalmers, 1992). By then, the founding members of the Cochrane collaboration had struggled for at least a decade with appraising the strength of evidence of the effectiveness of interventions in perinatal medicine. One quote summarizes well the focus of the collaboration: “Cochrane's and Poynard and Conn's emphasis on the need to summarize evidence derived from randomized controlled trials (as distinct from other kinds of evidence) is a reflection of the particular strengths of these formal clinical experiments for allowing inferences about the relative merits of different policies for disease prevention and treatment” (Chalmers et al., 1986).

Regardless of whether the Canadians, Americans, or British invented one of the “evidence‐based” permutations, the common denominator was a perceived need for more order among an overwhelming overload of information. An added concern, at least in U.K., was that a potential iceberg of unpublished data from clinical trials was being withheld from public scrutiny. This conspiracy theory prevails even today, with fingers pointing in all directions although predominantly towards the big pharma.

Oslo, Norway, is quite distant from the centres named above, and the evidence‐based movement would likely have passed unnoticed for some time if not Dr. Andy Oxman had moved directly from McMaster University to Oslo in 1994 to begin his work at the National Institute of Public Health in Norway. Soon after his arrival, the institute started organizing workshops on how to practice evidence‐based health care, and even a Nordic newsletter on EBH was launched (ISSN 0809‐5272). The National Institute of Public Health building was adjacent to the Faculty of Dentistry in Oslo, where we at the time had struggled for a decade with statistical analyses of heterogeneous clinical data from real‐life dentistry. (We are still striving!) The background was that Professor Ivar A. Mjör at the Institute for Dental Materials (NIOM) wanted to analyze accumulated data from a practice‐based research network (PBRN) among Nordic dental clinicians. Much energy was spent on rationalizing and testing statistical analyses and correct interpretation of the accumulated short‐term data versus long‐term data among an ocean of potentially explanatory variables and a range of more or less surrogate clinical outcomes. Several complex statistical approaches were attempted and discarded, including factor analyses, cluster analyses, principal component analyses, and classification and regression tree (CART) analyses. We ended up trying to explain the associations in “real‐life dentistry” by using multiple classification analysis of variance (MCA), Cox regression of survival data, and principal component analyses. Judged by the current literature, we still do not know today which statistics to apply to explain what works and what not of restorative and therapeutic care in everyday real‐life dental practice. Multilevel multivariate regression seems to become the most promising tool. I do not believe that data generated in RCTs in controlled environments by expert clinicians on highly selected study participants can be generalized to the daily situations of the ordinary general dentistry practitioner.

**Figure 1:**
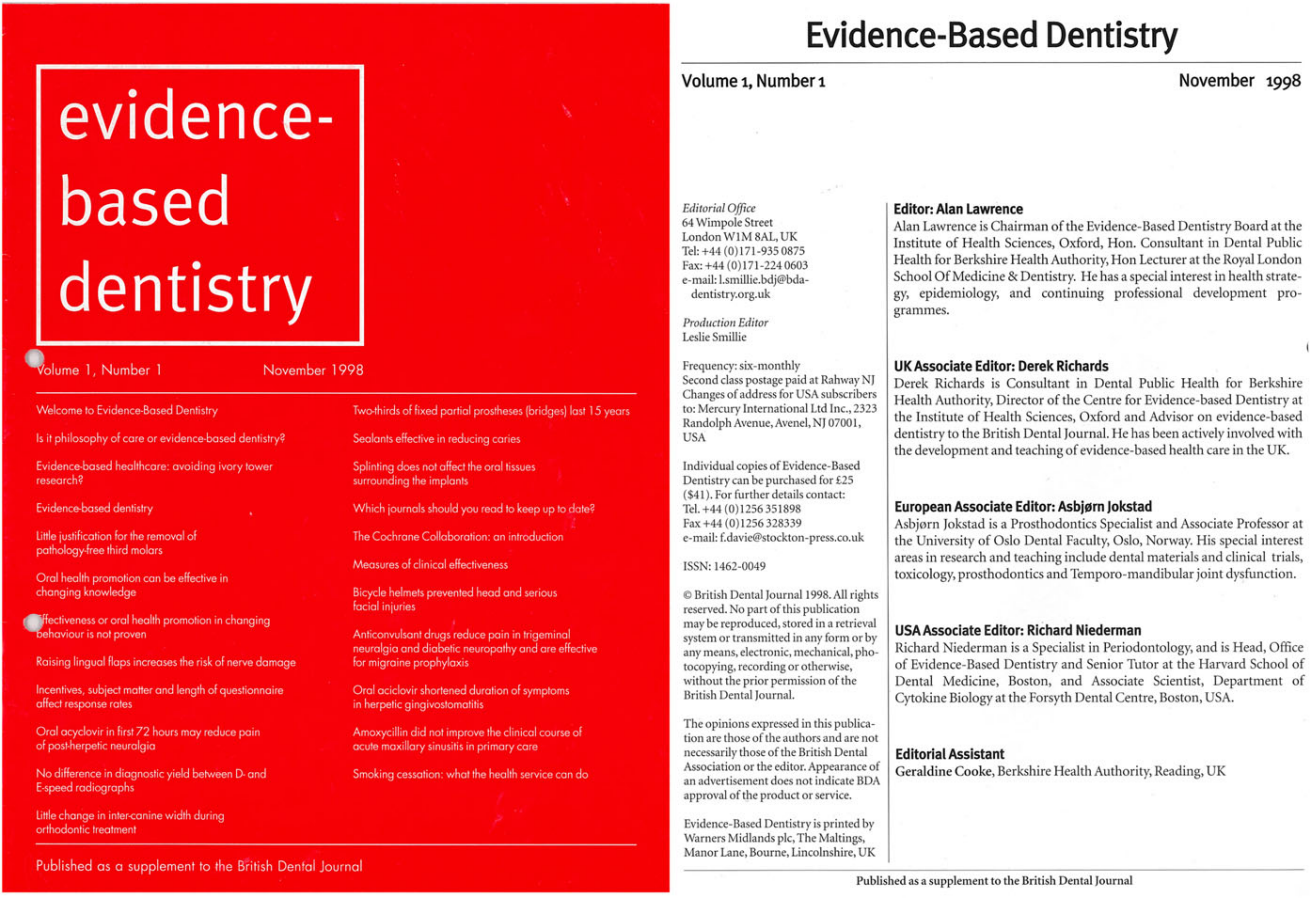
*Evidence‐Based Dentistry*, 1st issue, November 1998. URL: https://www.nature.com/ebd/

Also in U.K. in 1995, Dr. Alan Lawrence and Dr. Derek Richards established a *Centre for Evidence‐based Dentistry* in Oxford, UK (Richards & Lawrence, 1995), and they introduced the journal *Evidence‐Based Dentistry* with Alan as editor (Lawrence, 1998). Sadly, Alan passed away some years later, and the burden of editing the journal fell on Derek Richards, who deserves much praise for advancing EBD in many ways and for his extraordinary dedication to upholding the editorial standards of *Evidence‐Based Dentistry* (Figure 1). As Derek now steps down as editor after 20 years, he can look back on many outstanding editorials that are thought‐provoking for the reflective reader and the health professional dedicated to providing the best care and best practices (URL: https://www.nature.com/ebd/articles?searchType=journalSearch&sort=PubDate&type=editorial).

In the inaugural issue, I remarked that many stakeholders seemed to hesitate to start practicing EBD and offered some possible explanations (Jokstad, 1998). Today, most dentists must have heard about EBD (and if not, a visit to the website of the American Dental Association seems worthwhile: https://ebd.ada.org/en). Poor computer infrastructure, access, and skills are also no longer an excuse to not learn more about EB practice. The editorial ended with a perhaps naïve statement that “As the evidence‐based approach gains momentum, it is hoped that pertinent evidence can be generated through research. Perhaps someday there is enough group data to be particularized for individual‐centered health care in a meaningful manner.” However, even if we have better evidence today in many clinical areas about the effectiveness of interventions, the overwhelming focus is on the efficacy of a device, drug or biomaterial, and less on procedures and on the prevention of oral diseases. In sum, we still have wide gaps in our evidence basis for best management of the oral health of our patients.

Nevertheless, I am still adamant today as I was 20 years ago that “EBD is much more than RCT, and must always be regarded as an adjunct to, and not as a substitute for sound clinical judgement and patient preferences.” We need to be reminded that every patient encounter is a unique experience and requires an individualized approach. Moreover, we must recognize that knowledge is an educated interpretation of and not synonymous with information. As clinicians, we need to be able to present to our patients relevant evidence from science in combination with our clinical knowledge acquired through experience or education and strive for shared decision‐making. From this perspective of patient empowerment, I believe that EBD has contributed to improved oral health globally over the last two decades. I do hope that the patient empowerment will continue to improve further in the next 20 years. Happy anniversary, EBD!
